# Effectiveness of directly observed treatment short course (DOTS) on treatment of tuberculosis patients in public health facilities of Debre Tabor Town, Ethiopia: retrospective study

**DOI:** 10.1186/s13104-019-4424-8

**Published:** 2019-07-12

**Authors:** Chalachew Genet, Addisu Melese, Abebaw Worede

**Affiliations:** 1 Department of Medical Laboratory Sciences, College of Medicine and Health Sciences, Bahir Dar University, Bahir Dar, Ethiopia; 2 School of Medical Laboratory Sciences, College of Medicine and Health Sciences, University of Gondar, Gondar, Ethiopia

**Keywords:** TB, DOTS effectiveness, Debre Tabor

## Abstract

**Objective:**

The objective of this study is to assess effectiveness of directly observed treatment short course (DOTS) in treatment of tuberculosis (TB) patients in all public health facilities of Debre Tabor town, Ethiopia from January 2016 to December 2017.

**Result:**

Among 354 TB patients, 53.1% were males. Furthermore 22.6%, 40.4%, and 37% were smear positive pulmonary, smear negative pulmonary and extra pulmonary TB respectively. Study also revealed that TB–human immunodeficiency virus (HIV) co-infection and overall TB treatment success rate were 18.1% and 90.7% respectively. Regular weigh follow-up, sputum follow-up and HIV status were significantly associated with treatment success with P-value < 0.001, < 0.001 and 0.334 respectively. But TB treatment success weren’t associated with sex (P = 8.62), health facility type (P = 0.749) and TB type (P = 0.778). The study also showed that the overall TB treatment success rate was in line with World Health Organization (WHO) target on treatment success rate. Furthermore the study indicated higher TB–HIV co-infection and variations in conducting regular weight and sputum follow-up among HFs.

## Introduction

Tuberculosis (TB), one of the oldest infectious diseases, is causing significant morbidity and mortality all over the world [1]. Despite the availability of effective anti TB drugs, it is one of the top 10 causes of death worldwide. Based on World Health Organization (WHO) report, there are 10 million cases and 1.6 million deaths globally in 2017 [1]. Among the total death occurred in the same year, more than 95% death occurred in low and middle income countries [1].

The impact of tuberculosis is more pronounced in developing than developed world mainly in Africa where on average 281 cases per 100,000 population is reported in 2014 which is twice of the global average (133/100,000 population) on the same year [2].

As part of developing country, Ethiopia is highly affected by the impact of tuberculosis. Ethiopia is one of the 22 TB high burden countries. Based on 2016 WHO report, the incidence of TB cases in Ethiopia were 207 per 100,000 population in 2014 [2]. Moreover TB is the second cause of death in Ethiopia based on 2009/10 health and health related indicators of the Federal Ministry of Ethiopia [3].

By recognizing the global impact of tuberculosis as well as non-organized and variability in tuberculosis control program, WHO developed a strategy called directly observed treatment short course (DOTS). The DOTS strategy have five fundamental components: sustained political and financial commitment; case detection through quality ensured bacteriology; standardized treatment with supervision and patient support; effective drug supply and management system as well as proper monitoring and evaluation system [4]. Ethiopia launched the DOTS strategy since 1992 as part of National Tuberculosis and Leprosy Control Program (TLCP) to treat TB patients [5, 6].

Effectiveness of DOTS with respect to treatment outcome is reported in different parts of the world [6–12]. But there is limited study conducted to assess the effectiveness of DOTS in all public health facilities of Debre Tabor town at a time. Thus this study was conducted to assess the effectiveness of DOTS on the treatment of TB patients in all public health facilities of Debre Tabor town in Amhara regional state of Ethiopia.

## Main text

### Study design and setting

A retrospective cross sectional study was conducted in all four public health facilities (HFs) of Debre Tabor Town, Ethiopia from January 2016 to December 2017. Among four public HFs, three of them were Health Centers (HCs) named Hidar 11 HC, Debre Tabor Health Center (DTHC) and Ginbot 20 HC as well as the fourth HF is Debre Tabor Hospital (DTH). In all HFs, TB diagnosis, treatment and prevention service is given. TB is diagnosed in all governmental health facilities studied using clinical data, X-ray and sputum microscopy by taking two sputum samples in spot–spot combination. Irrespective of their HIV status, all TB patients will take the same drug during their course of TB treatment for different duration depending on the type of TB. The study area, Debre Tabor town, is located 100 km and 666 km away from regional capital Bahir Dar and country capital Addis Ababa respectively. Based on 2007 E.C Central Statistics Agency (CSA) report, the town has a total population of 55,596.

### Data collection

Data were collected by 2 Bachelor of Science (BSc.) holder Medical laboratory workers using checklist prepared for this purpose based on the contents found on the TB registration book. Some of the data collected include age, sex, patient address, contact person information, types of TB, patient category, HIV test and others. Record of all 354 TB patients who attended their treatment from January 2016 to December 2017 was reviewed to assess the effectiveness of DOTS in the treatment of TB patients.

### Data analysis

The collected data was labeled and coded. It was entered and analyzed using SPSS. Different statistical analysis like frequency, percentage, Pearson’s Chi square test and Fisher’s exact test were done.

### Results

Among 354 TB patients included in the study, 188 (53.1%) of them were males and 166 (46.9%) were females. The study also indicated that 37% of TB patients were in the age group of 21–30 years and 85.6% TB patients were new cases (Table 1).

**Table 1:** Basic characteristics of TB patients in public health facilities of Debre Tabor Town, 2018

Variables	DTH: No (%)	DTHC: No (%)	Hidar 11 HC: No (%)	Ginbot 20 HC: No (%)
Sex				
Male	78 (55.3)	59 (48.4)	32 (58.2)	19 (52.8)
Female	63 (44.7)	63 (51.6)	23 (41.8)	17 (47.2)
Age (years)				
< 10	3 (2.1)	2 (1.6)	7 (12.7)	0 (0)
11–20	28 (19.9)	25 (20.5)	6 (10.9)	3 (8.3)
21–30	51 (36.2)	42 (34.4)	21 (38.2)	17 (47.2)
30–40	24 (17)	18 (14.8)	5 (9.1)	4 (11.1)
> 40	35 (24.8)	35 (28.7)	16 (29.1)	12 (33.4)
Patient category				
New case	112 (79.4)	107 (87.7)	49 (89.1)	35 (97.2)
Re-treatment	21 (14.9)	8 (6.6)	0 (0)	1 (2.8)
Transfer in	8 (5.7)	6 (4.9)	6 (10.9)	0 (0)
Failure	0 (0)	1 (0.8)	0 (0)	0 (0)

*DTH* Debre Tabor Hospital, *DTHC* Debre Tabor Health Center, *Hidar 11 HC* Hidar 11 Health Center, *Ginbot 20 HC* Ginbot 20 Health Center, 303

**Fig. 1:**
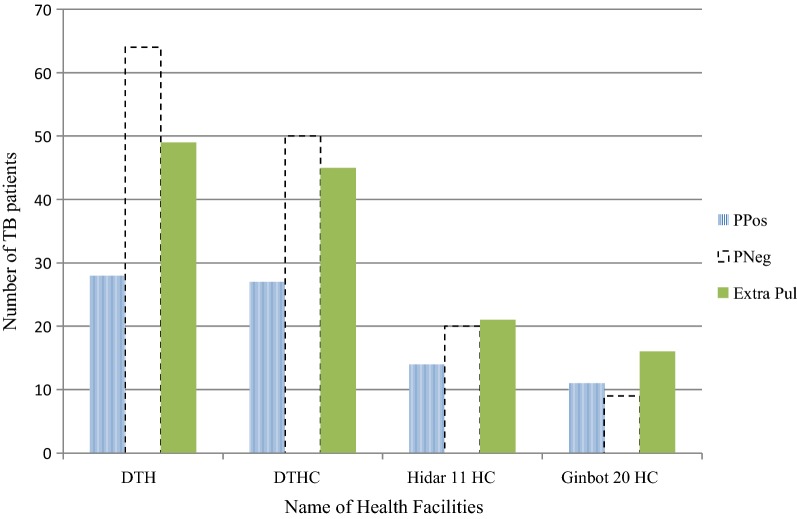
Type of TB patients in all public health facilities of Debre Tabor town, 2018. *DTH* Debre Tabor Hospital, *DTHC* Debre Tabor Health Center, *Ginbot 20 HC* Ginbot 20 Health Center, *Hidar 11 HC* Hidar 11 Health Center, *PPos* smear positive pulmonary TB, *PNeg* smear negative pulmonary TB, *Extra Pul* extra pulmonary TB

The study also indicated that from the total 354 TB patients included, 80 (22.6%), 143 (40.4%) and 131 (37%) were diagnosed as having smear positive pulmonary TB, smear negative pulmonary TB and extra pulmonary TB patients respectively. Among HFs studied, the highest smear positive pulmonary TB patients were identified in Ginbot 20 HC followed by Hidar 11 HC with 30.6% and 25.5% respectively (Fig. 1).

The highest number of cured TB patients was reported in Ginbot 20 HC followed by DTH (Table 2).

**Table 2:** Treatment outcome of TB patients in public HFs of Debre Tabor Town, 2018

HFs (N = 354)	Treatment outcome: No (%)				
	Cured	Complete	Died	Failure	Not indicated
DTH (N = 141)	28 (19.9)	99 (70.2)	8 (5.7)	3 (2.1)	3 (2.1)
DTHC (N = 122)	24 (19.7)	88 (72.1)	7 (5.8)	1 (0.8)	2 (1.6)
Hidar 11 HC (N = 55)	10 (18.2)	41 (74.6)	3 (5.4)	0 (0)	1 (1.8)
Ginbot 20 HC (N = 36)	8 (22.2)	22 (61.1)	3 (8.3)	1 (2.8)	2 (5.6)

*HFs* health facilities, *DTH* Debre Tabor Hospital, *DTHC* Debre Tabor Health Center, *Hidar 11 HC* Hidar 11 Health Center, *Ginbot 20 HC* Ginbot 20 Health Center

Generally similar treatment success rate of TB patients was observed in all HFs assessed except Ginbot 20 HC. Moreover the present study also indicated that 320 (90.4%) of TB patient treatment was successful. With no significant (P = 0.749) association, TB patients who attended their treatment in HCs showed almost similar treatment success rate (91.1) with those patients who attended their treatment in hospital (90.1%).

The study also showed that Hidar 11 HC and DTHC performed regular weight and sputum follow-up in 63.3% and 77.8% of TB patients attending their treatment respectively which is higher than other health HFs of the present study. The study also indicated that TB patients with regularly weight follow-up done (P < 0.001) and sputum follow-up done (P < 0.001 (for pulmonary positive TB patients)) had higher treatment success with 100% and 100% respectively than those TB patients with irregular weight and sputum follow-up done.

Furthermore the study also indicated that almost all TB patients (98.9%) were tested for HIV where 18.1% of them found to be positive. Those patients who were HIV negative have better treatment success rate than those TB patients who were HIV positive with treatment success rate of 91.4% and 87.5% respectively. The association was not statistically significant (P = 0.334). Moreover the study also indicated that higher numbers of HIV positive TB patients were attending their TB treatment in hospital than HCs with 25.5% and 12.2% respectively. This association was statistically significant (P < 0.001).

### Discussion

Majority of TB cases (85.6%) were new cases which were comparable with a study done in Addis Ababa, Ethiopia and Debre Tabor, Ethiopia which reported 88.9% [12] and 84.5% [13] respectively. On the other hand the present study finding was lower than study done in HCs of Mekelle town, Ethiopia [6]. This variation with a study in Mekelle might be explained that the present study included HCs and hospital as well as study time gap where the study in Mekelle was conducted 8 year prior than the present study. The study time gap difference might increase chance of antibiotic resistance *Mycobacterium tuberculosis* emergency which might contribute in increasing the re-treatment cases.

The study also indicated that 37%, 40.4% and 22.6% of TB patients were diagnosed as having extra pulmonary TB (EPTB), smear negative pulmonary TB (PTB−) and smear positive pulmonary TB (PTB+) respectively. Comparable finding was reported in Debre Tabor general hospital, Ethiopia which was conducted taking 9 year data from single hospital from 2008 to 2016. The study reported that 36.9%, 36.9% and 24.5% of TB patients as having EPTB, PTB− and PTB+ respectively [13]. But this finding was in contrary with a study done in Mekelle town, Ethiopia [6]. This variation might be explained that unlike present study which includes TB patients diagnosed in hospital, the study in Mekelle included only TB patients diagnosed in HCs where there might be limited laboratory diagnostic services to detect etiological agent of TB compared with laboratory diagnoses services in hospital which will further increase the figure of extra pulmonary and smear negative pulmonary TB patients.

On the other hand, the present study which indicated 63% TB patients as having pulmonary TB was related with a study done in all regions of Ethiopia [8]. A study done in Dilla of Southern Ethiopia [7] indicated that 35.4% of TB patients were diagnosed as having PTB+. This finding showed higher PTB+ result compared with the present study of 22.6% PTB+. This variation might be explained by presence of better laboratory diagnostic service in the study done in Dilla since it is a referral hospital unlike the present study which is conducted in general hospital and HCs.

This study also revealed that 18.1% of TB patients were HIV positive. This finding was in agreement with study done in Mekelle town of Ethiopia where 15.4% of TB patients tested for HIV were positive [6]. Similarly the result was also comparable with study done in North Gondar zone prison, Ethiopia which indicated 12.4% [11]. With regard to HIV testing, the present study indicated that 98.9% of TB patients were tested. This result was in agreement with one of WHO indicator of stop TB strategy which states 100% documentation of HIV status among TB patients [14].

The 88.8% cure rate among smear positive pulmonary TB patients was similar with study done in Ethiopian Somalia regional state which was 84% [10]. On the other hand present study cure rate was higher than study done in Nigeria which reported cure rate of 76.6% [9]. This variation might be explained by study time gap, difference in sample size and study setting.

The 90.7% treatment success rate of the present study agrees with WHO recommended target value of treatment success rate > 90% which is one of the ten indicators set to monitor the implementation of end TB strategy up to 2025 [14]. Similarly treatment success rate of the present study was supported by different studies conducted in Ethiopia with treatment success rate of 82.7% in Addis Ababa [12], 86.8% in Ethiopian Somalia [10] and 90.1% in Northwest Ethiopia [13].

On the other hand present study treatment success rate (90.7%) was higher than a study done in different parts of Ethiopia with treatment success rate of 78.7% in Bahir Dar, Ethiopia [15] and 84% in Mekelle, Ethiopia [6]. This difference might be due to small sample size used in both studies compared with present study as well as study time gap.

Similarly treatment success rate of TB patients in the present study (90.7%) was much higher than study done in all regions of Ethiopia by taking representative HFs which reported an average treatment success rate of 55.8% [8]. This variation might be explained duet to sample size variation, study setting difference, HF type difference as well as high transfer out rate in study done in all regions which was 26.2%.

### Conclusions

The overall treatment success rate of TB patients was 90.7%. This was in line with WHO target on treatment success rate. The study also indicated that significant proportion (18.1%) of TB patients were HIV positive. Moreover regular sputum follow-up and weight follow-up were significantly associated with treatment successes. Thus we recommend the responsible officials working in health offices and health bureau to work in sustaining this treatment success rate. Moreover attention shall be given in conducting regular sputum follow-up and weight follow-up test regularly.

## Limitations

Since the quality of the data is highly dependent on the proper recording of the values to the TB registration book by health professionals working in TB clinic, it can be affected by registration quality.

## Data Availability

All data collected are included within this study.

## Abbreviations

ACF: acid fast bacilli
CSA: Central Statistics Agency
DOTS: directly observed treatment short course
DTH: Debre Tabor Hospital
DTHC: Debre Tabor Health Center
EPTB: extra pulmonary TB
HCs: Health Centers
HFs: health facilities
HIV: human immunodeficiency virus
PTB−: smear negative pulmonary TB
PTB+: smear positive pulmonary TB
TB: tuberculosis
TLCP: National Tuberculosis and Leprosy Control Program
WHO: World Health Organization
